# Unravelling the challenges of dementia home care in India

**DOI:** 10.1002/alz.085019

**Published:** 2025-01-09

**Authors:** Jayashree Dasgupta

**Affiliations:** ^1^ Samvedna Senior Care Foundation, Gurugram India

## Abstract

**Background:**

India has a mixed public‐private healthcare system however most care is out‐of‐pocket. Despite projections that the older population is expected to rise from current 138 million to 194 million by 2031, services catering to the needs of people living with dementia remain limited. Ailing family members and older adults have traditionally been cared for in an intergenerational joint family system where members reside together. However, social transitions, including more women entering the workforce and migration, both within India and international migration, for better job opportunities, have led to changes in this traditional family system. This presents significant challenges for families as they end up providing majority of dementia care with limited professional support, limited knowledge and virtually no access to training, counseling and other support services.

**Methods:**

Using a case study approach we describe the complexity of issues faced by Indian families. Cases were retrospectively sampled from families that availed services from an eldercare organization providing a range of services in Gurugram India, between Jan 2018 to March 2023. Using this approach, we specifically draw attention to 1) challenges faced by transnational Indian families when parents struggle to live independently 2) challenges faced by an only child due to limited dementia specific services 3) Dynamics within families as they navigate various aspects of decision making, finances and family relocation for dementia care.

**Results:**

This presentation will highlight important themes that emerge and focus on areas which need to be addressed whilst developing homecare services for dementia in India. In particular, we highlight the need for professionally delivered family support, eldercare services tailored to dementia, training for a dementia ready work force in India, and the role that technology plays in supporting dementia care.

**Conclusions:**

This qualitative and experiential account provides impetus for further work to develop culturally appropriate strategies to support dementia care in India.

